# African American College Students’ Contextual Emotion Expression

**DOI:** 10.1007/s42761-024-00286-6

**Published:** 2025-01-30

**Authors:** Deon W. Brown, Fantasy T. Lozada, Zewelanji N. Serpell, Vivian A. Dzokoto, Julie C. Dunsmore

**Affiliations:** 1 Department of Psychological, Health & Learning Sciences, University of Houston, Steven Power Farish Hall, Houston, TX 77004 USA; 2 Department of Psychology, Virginia Commonwealth University, 808 W. Franklin St, Box 842018, Richmond, VA 23284 USA

**Keywords:** Emotion expression, Context, Racial discrimination, Black, College campus

## Abstract

The current study aimed to explore patterns of self-reported emotion expression across familial and college campus contexts among African American college students and the associations of these patterns with contextual features on college campuses (i.e., racial demographics of the university overall and students’ friend group and racial discrimination experiences). Theoretical support included Triple Quandary Theory and emotional development models. Online survey data from 169 self-identified African American college students (62.4% female; *M*_age_ = 20.60) across three college campuses were analyzed to compare reports of emotion expression with family members and emotion expression on campus. Additionally, family and campus emotion expression reports were analyzed via latent profile analysis (LPA) to identify latent profiles of emotion expression and their predictors. Four profiles of emotion expression across familial and college campus contexts emerged: *Positive Low Expressors* (*n* = 55; 32%), *High Family – Low Campus Expressors* (*n* = 44; 26%), *Consistent Expressors* (*n* = 37; 22%), and *Low Family Positive – Consistent Negative Expressors* (*n* = 33; 20%). College campus type was not a significant predictor of profile membership. However, campus experiences of racial discrimination and racial composition of friend group were significant predictors. African American college students’ varying emotion expression in familial domains relative to public domains (i.e., college campuses) may reflect variation in their racial experiences. These findings have implications for a contextual based understanding of African Americans’ emotional competence, broadly, and the necessity for colleges to consider how campus experiences shape African American college students’ emotional functioning.

Within the USA, African Americans remain a numerical and ethnic minority. Their current experiences are rooted in a history of enslavement, persecution, oppression, and discrimination. Over time, these experiences have yielded beliefs and behaviors among African American groups that are adaptive in preparing for and responding to discriminatory experiences and intergroup interactions (Boykin & Toms, 1985; García Coll et al., 1996). García Coll and colleagues (1996) assert that adaptive cultural practices arise out of racism and subsequent promoting and inhibiting environments. These conditions have implications for ethnic-racial minorities’ familial socialization processes and emotional developmental competencies, including emotional flexibility or code-switching (Lozada, Jagers et al., 2022). While recent theory has emerged to describe African American parents’ socialization of emotion-related behavior in response to the experience of racial discrimination (e.g., Dunbar et al., 2017), promoting strategies such as emotional vigilance and emotion suppression, there remains little understanding and empirical investigation of African Americans’ emotional development beyond the familial context. The purpose of the current study was to investigate the contextual differences in emotion expression among African Americans as a demonstration of how emotional competence among this ethnic-racial group may reflect cultural adaptation in the face of various contextual exposure (e.g., ethnic-racial groups and racial discrimination experiences) across familial and public contexts.

## Triple Quandary Theory and African American Contextual Emotion Expression

Triple Quandary Theory (TQT) asserts that African Americans constantly navigate three distinct psychological spaces: the mainstream cultural experience, the Black^1^ cultural experience, and the minority cultural experience (Boykin & Toms, 1985). The mainstream cultural experience reflects US American society of which all citizens participate in, regardless of background or identity. US citizens, including African Americans, participate in and are socialized into mainstream culture through work, educational, media, judicial, and consumer systems, all of which traditionally reflect white, Eurocentric values (e.g., individualism, conformity, and universality). The Black cultural experience reflects Western African traditions and cultural values (e.g., communalism, spirituality, and importance of affect) that have endured and been transformed through the necessity of navigating an American system that has promoted and propagated assimilation, racism, and oppression. African Americans learn Black culture as styles of behavior through their interactions with parents, other family members, and with other African Americans (e.g., patterns of communalistic or spiritual coping and open emotion expression; Lozada, 2024). The minority cultural experience reflects the necessary coping and adaptation orientations to racism and oppression that African Americans use to navigate racial discrimination, bias, and marginalizing experiences (e.g., engaging in behaviors to avoid experiencing bias such as adopting identity shifting or assimilating behaviors or suppressing overt emotional expression in certain contexts; Loyd et al., 2023; Lozada, Riley et al., 2022). African Americans most often learn how to navigate discriminatory experiences through familial socialization (e.g., Hughes et al., 2006) but may also learn coping and adaptation through exposure and shared experiences with members of other minoritized groups. The mainstream experience, the Black cultural experience, and the minority experience present a “triple quandary” for African Americans in which they must operate in different psychological spaces to achieve success in America.

Researchers have theorized that African Americans’ navigation of these multiple cultural contexts and the minoritizing experiences of racialized emotion (e.g., stereotypes of the “angry Black person,” outgroup misrecognition of African American emotion expression; Durik et al., 2006; Halberstadt et al., 2013, 2018) has promoted awareness and incorporation of emotion norms in different spaces (i.e., public versus private contexts and mainstream versus Black contexts; Dunbar et al., 2017; Lozada, Jagers et al., 2022). Yet, researchers have largely conducted studies on African Americans’ emotion expression in discrete spaces (see Labella, 2018 for a review of familial expression among parental figures). One exception was a study that asked multi-ethnic-racial college students (including African Americans) to recall emotion expression in the familial context from early childhood (Morelen et al., 2013). Researchers found differences in remembered childhood family emotion expression between White, Asian, and African American students, but all ethnic-racial groups were similar in that students remembered their families expressing more positive emotion than negative emotion. Studies of African Americans’ emotion expression in public contexts are more limited and have presented African American college students with scenarios of imagined emotional reactions. These studies found that African Americans express positive emotion more than negative emotion in public contexts, particularly when the context is predominantly White (Vrana & Rollock, 1996, 1998, 2002). Taken together, these studies suggest that African Americans express more positive emotion than negative emotion overall and that contextual features such as the racial demographics of a setting can influence African Americans’ emotion expression.

While considering literature on African Americans’ emotion expression in terms of familial and public contexts may be helpful for organizing the results of previous studies, such an approach may oversimplify the construct of emotion. Emotion is a dynamic construct, meaning that it is fluent and contextually bound (Mesquita & Boiger, 2014). It is difficult for researchers to capture based on static measures of emotion that only reflect one context. This may be especially the case for African Americans, who value development of emotional flexibility or code-switching to survive and thrive with contextually-adaptive expression (Lozada, Jagers et al., 2022). Thus, systematic investigation of emotion expression across contexts may lead to increased understanding of African Americans’ emotion expression. Furthermore, the current study takes a person-centered approach. Rather than examining relations between variables across individuals, a person-centered approach identifies groups of individuals who share similar within-person patterns of variables (Laursen & Hoff, 2006). This provides a holistic view of relative consistency in emotional expression across contexts or distinction in emotional expression between contexts within individuals. Consistent with Triple Quandary Theory, using a person-centered approach recognizes that African Americans may vary in the styles or patterns they use to manage emotional expression across contexts while still maintaining a sense of emotional authenticity as a person (Boykin & Toms, 1985; Lozada, Jagers et al., 2022). The current study attempted to capture nuanced patterns of African Americans’ emotion expression using latent profile analysis (LPA), a person-centered analytic approach, with participants’ responses on a validated measure of emotion expression modified to account for the public context in addition to the familial context.

## Current Study: Emotion Expression Across Contexts Among African American College Students

The current study aimed to investigate the contextual influences of emotion expression among African American college students through a person-centered approach. As a first exploration of contextual influence, we aimed to understand how African American college students self-report their emotion expression across contexts. Consistent with scholars’ previous articulation of the importance of considering private (i.e., familial) versus public contexts for African Americans’ emotion-related behavior (e.g., Dunbar et al., 2017), we examined how African American college students reported their expression of positive and negative emotions in the private familial setting and the relatively more public, college campus setting. We also wanted to examine whether the variation of public and private emotion expression yielded distinct profiles of contextual emotion expression.

Consistent with previous research on African American familial and public emotion expression, we expected distinct profiles of college students’ positive and negative emotion expression across family and college campus contexts to emerge (hypothesis 1). We expected at least one profile to emerge that is characterized by higher levels of positive expression than negative emotions based on previous patterns of emotion expression in the family context (Hill & Tyson, 2008; Nelson, O’Brien, Calkins et al., 2012). While we do not make specific predictions about other profiles across contexts, we believe it is important to investigate profiles based on public contexts as well as family contexts because they are salient for African Americans’ emotional messaging and may reveal additional patterns of emotion expression to those of parental figures. Further, we sought to examine an additional influence of context relevant to the African American psychological experiences of the “triple quandary.” Guided by Triple Quandary Theory (Boykin & Toms, 1985), we sampled African American college students at three universities which varied in their racial demographics to examine whether contextual emotion expression profile membership differed as a function of campus racial demographics: whether students attended a predominantly White institution (PWI; representing a mainstream cultural context), a Historically Black College/University (HBCU; i.e., representing a Black cultural context), and a relatively racially diverse college in which over half of the student body identified as an ethnic-racial minority (i.e., representing a minority cultural context). Given previous scholars’ discussion about the role of racial discrimination in shaping emotion-related behaviors among African Americans (Dunbar et al., 2017; Lozada, Jagers et al., 2022), we also examined racial discrimination as a predictor of profile membership. Furthermore, we hypothesized that racial demographics (hypothesis 2a) and racial discrimination (hypothesis 2b) were factors that would account for patterns of African American college students’ emotion expression in the family and campus contexts. This hypothesis was exploratory given the limited amount of literature that explores connections between these factors and emotion expression that informs our ability to predict directions. Lastly, we explored the role of gender and racial composition of friend group on campus in African American students’ profiles of emotion expression.

## Method

### Participants

Participants were 169 college students ranging from 18 to 54 years old (*M*_*age*_ = 20.60); 144 indicated that they were African American/Black and 25 said they were multi-racial. Regarding gender, 62.7% were female, 35.5% were male, and 1.8% were non-binary or transgender. Students ranged in their classification: 33.7% freshman, 24.3% sophomore, 22.5% junior, 18.9% senior, and 0.6% other. Ten students reported full-time employment at the time of the study, 61 indicated that they worked part-time, and 32 said they were unemployed. Student representation at each college campus racial demographic type was as follows: 24.3% at a PWI, 53.8% at a relatively racially diverse college, and 21.9% at a HBCU.

### Procedures

Participants were recruited from three colleges in the Southeastern United States via email through Black college student organizations, advertisement through psychology courses, an undergraduate research management system, and flyers with QR codes posted at various locations on each campus. Participants accessed the online survey programmed in the online survey platform, Qualtrics, via a link from the recruitment email or from a flyer QR code. The online survey included a consent form before allowing participants to access the questionnaire and provide a means of contact to receive either a $10 Amazon gift card or university credit for participating in the survey. Participants had to be at least 18 years of age and self-identify as African American/Black in order to participate in the study.

### Measures

#### Emotion Expression

Emotion expression with family and others on campus was assessed using a modification of the Self-Expressiveness in the Family Questionnaire (SEFQ; Halberstadt et al., 1995) short form. The SEFQ is a self-report measure of the frequency of an individual’s positive and negative emotion expression within the family. The short form includes 29 items rated on a 9-point Likert scale (i.e., 1 = not at all frequently and 9 = very frequently) and yields three subscales: positive emotions (“Praising someone for good work,” 11 items, for this sample, *α* =.88), negative dominant emotions (“Showing contempt for [making fun of] another’s actions,” 8 items, *α* =.78), and negative submissive emotions (“Expressing disappointment over something that didn’t work out”; 8 items, *α* =.75). Our modification included altered instructions so that participants also reported the frequency that they engaged in specific emotion expression behaviors with “others on campus.” Wording for six items was slightly altered to be appropriate for both family and campus contexts (i.e., “family member” was replaced with “person” or “someone”). Internal consistencies for the three subscales were satisfactory (positive emotions, *α* =.86; negative dominant emotions, *α* =.82; negative submissive emotions, *α* =.78). Mean scores were computed for positive, negative dominant, and negative submissive emotion expression subscales for both family and campus contexts. Higher scores on each subscale represented more frequent emotion expression. The original SEFQ has been previously used to assess familial emotion expression among African American samples and has demonstrated acceptable inter-item reliability and convergent validity with other indicators of emotion-related beliefs and behavior (Brown et al., 2015; Halberstadt et al., 2013; McCoy & Raver, 2011).

#### College Campus Racial Composition Type

Participants were asked to report which college they attended out of the three data collection sites. Each university was assigned a college racial composition type based on the public racial demographic statistics for each school. Specifically, a school was identified as a PWI when White students represented more than 50% of the student enrollment of the campus. A school was identified as racially diverse if White and Black students each represented less than 50% of the student enrollment of the campus. A school was identified as a HBCU if Black students represented 60% or more of the student enrollment of the campus and it was established before 1964 with the purpose of educating African American students (according to the Higher Education Act of 1965; Samuels, 2010). University 1 had a student enrollment of 4.5% African American students, 28.6% students from other racial/ethnic or multi-racial backgrounds, and 66.9% White students; University 1 was classified as a PWI. University 2 had a student enrollment of 18.7% African American/Black students, 38.2% students from other racial/ethnic or multi-racial backgrounds, and 43.1% White students; University 2 was classified as a racially diverse college. University 3 had a student enrollment of 95% African American/Black students, 4.07% students from other racial/ethnic or multi-racial backgrounds, and 0.93% White students; University 3 was classified as a HBCU.

#### Racial Discrimination Experiences

Racial discrimination was assessed using items from the Black Male Experiences Measure (BMEM; Cunningham & Spencer, 1996). The BMEM was originally designed to assess Black males’ experiences and perceptions in public settings. It originally consisted of three subscales: proximal negative experiences, distal negative experiences, and negative inference experiences (*α* =.91 for total scale). We consulted authors to modify the BMEM to be used with African American college students regardless of their gender identification given its emphasis on perceptions of behaviors in the public context (see Corprew & Cunningham, 2012, for example with an adolescent sample). For the current study, we only used items from the negative youth experiences subscale. Participants reported on the frequency (i.e., “how often”) of personal experiences as members of their racial/ethnic group on campus. Instructions did not specify a timeframe. Sample items included “professors think you have plagiarized or cheated on your class assignment” and “campus police thought that you were doing something wrong.” Participants responded using the following scale: 1 = never (zero times), 2 = almost never (one to three times), 3 = sometimes (four to six times), 4 = almost always (seven to nine times), or 5 = always (ten plus times). A mean score was computed across the five items, with higher scores indicating more frequent experiences with racial discrimination on campus (*α* =.75).

#### Exploratory Variables

To be used as potential covariates of profiles of emotion expression, participants reported on their gender identification and the racial composition of their friend group on campus. With regard to the racial composition of the friend group on campus, participants were asked to reflect on their friends on campus (both those that they were “emotionally close with” and those they “hang out with”) and report the number of black people on a 5-point scale from 1 = almost all Black people to 5 = almost all people of other races. Higher scores indicated a friend group with more non-Black friends.

## Results

### Preliminary Analyses

Descriptive statistics of the study variables overall and by school are presented in Table 1. No significant mean differences emerged across universities for any study variables (ps > 0.05). Bivariate correlations of study variables are presented in Table 2.

**Table 1:** Descriptive Statistics of Study Variables (*N* = 169)

Variable	Overall		University 1		University 2		University 3	
	*M*	*SD*	*M*	*SD*	*M*	*SD*	*M*	*SD*
Racial Composition of Friends	2.33	1.21	2.62	1.39	2.44	1.12	1.28	0.57
Racial Discrimination	2.24	0.39	2.24	0.39	2.20	0.39	2.17	0.36
Family Positive Emotion Expression	5.73	1.64	5.85	1.58	5.56	1.75	5.94	1.49
Family Negative Dominant Emotion Expression	3.51	1.56	3.67	1.81	3.70	1.50	3.13	1.22
Family Negative Submissive Emotion Expression	4.29	1.61	4.35	1.60	4.24	1.46	4.60	2.13
Campus Positive Emotion Expression	4.77	1.58	5.02	1.56	4.63	1.56	4.55	1.62
Campus Negative Dominant Emotion Expression	3.08	1.49	3.24	1.58	3.11	1.49	2.71	1.32
Campus Negative Submissive Emotion Expression	3.30	1.50	3.46	1.54	3.23	1.50	3.25	1.81

**Table 2:** Bivariate Correlations of Study Variables (*N* = 169)

	1	2	3	4	5	6	7	8
1. Racial Composition of Friends	−	.07	−.22**	−.12	−.13	.08	.08	.13
2. Racial Discrimination		−	.05	.11	.15	.17*	.18*	.11
3. Family Positive Emotion Expression			−	.26**	.55**	.38**	−.04	.13
4. Family Negative Dominant Emotion Expression				−	.47**	.15	.47**	.30**
5. Family Negative Submissive Emotion Expression					−	.32**	.17*	.58**
6. Campus Positive Emotion Expression						−	.42**	.55**
7. Campus Negative Dominant Emotion Expression							−	.51**
8. Campus Negative Submissive Emotion Expression								−

*p < 0.05, **p < 0.01

Overall, students’ friend group on campus consisted of mostly Black people (*M* = 2.33) and students experienced racial discrimination on campus about two to three times (*M* = 2.24). Additionally, students reported somewhat frequent positive emotion expression in the family (*M* = 5.73) and relatively less frequent emotion expression otherwise. Students’ experiences of racial discrimination on campus were significantly related to their expression of positive emotion (*r* =.17, *p* =.03) and negative dominant emotion (*r* =.18, *p* =.02) in the campus context. Racial demographics of students’ friends on campus was only significantly related to students’ expression of positive emotion in the family (*r* = −.22, *p* <.01) but was unrelated to any other variable of interest. SEFQ subscales were largely correlated with each other. To examine the association of gender with study variables of interest, independent samples *t*-tests were conducted. Given the low number of non-binary or transgender students in the study, only those who self-identified as a male or female were analyzed. Male and female students did not differ significantly on any of the study variables of interest (*p*s >.05). Given the general lack of significant association with emotion expression variables, gender was not used as a covariate in the latent profile analyses below.

### Exploratory and Primary Analyses

To examine the existence of profiles of African American/Black college students’ emotion expression at home and on campus and to test whether campus racial composition type, racial discrimination, and racial composition of friend group were predictors of emotion expression profiles, we used Vermunt’s 3-Step LPA approach via Latent Gold 5.1 (Vermunt, 2010; Vermunt & Magidson, 2013). Vermunt’s 3-Step approach is an attempt to correct classification errors that introduce bias when trying to assess the relationship between class membership and external variables. The Step3 module was advantageous for the current study because the indicator variables for class membership were continuous. Thus, the first step of LPA was to enter all emotion expression variables as continuous indicators in the latent class model to estimate the number of latent profiles.

Next, multiple models were estimated to compare profile solutions. Model selection was based on a mixture of statistics representing the information criterion (IC) method (i.e., Bayesian information criterion [BIC], Akaike information criterion [AIC], and Akaike information criterion 3 [AIC3]), local goodness-of-fit tests (i.e., the maximum bivariate residual [BVR]), the ability to distinguish classes (i.e., entropy), and model fit in comparison to the previous model solution (i.e., bootstrap likelihood-ratio test [BLRT] p-value; Bauer, 2022; Magidson et al., 2020; Salas-Wright et al., 2023; Tein et al., 2013). Lower values for IC statistics and maximum BVR indicate better model fit (Ortiz et al., 2024). BVR values less than 4 indicate a significant amount of residual association explained (Magidson et al., 2020). Acceptable entropy values are .80 or higher, with higher values closer to 1 indicating better model accuracy in defining profiles (Salas-Wright et al., 2023). Finally, significant BLRT *p*-values (*p* <.05) indicate that the current model demonstrates significantly better model fit than the preceding model (Tein et al., 2013). Model estimation went up to and stopped at 10 profiles because the BLRT did not demonstrate significant model improvement after two subsequent models.

Fit statistics for the 10 profile solutions are presented in Table 3. Entropy values for all models were acceptable. The IC method suggested that a 4-profile solution or 8-profile solution fit the data best because the BIC value was lowest for the 4-profile solution and the AIC and AIC3 statistics were lowest for the 8-profile solution. Further, the 8-profile solution had a BVR value below 4. Examination of the profiles represented in the 8-profile solution revealed that at least two of the profiles in this model were difficult to interpret and consisted of less than 5% of the sample. Given this, we returned to the 4-profile solution but corrected for the large BVR (> 4) found between campus negative dominant expression and campus positive expression and campus negative submissive expression and campus positive expression. A direct effect accounting for the residual between these variable pairs was applied (see Lozada, Jagers et al., 2022 for previous use of this BVR correction), providing for a more parsimonious model with a better model fit than the 8-profile solution across all IC statistics and with a lower maximum BVR and an acceptable entropy. This 4-profile solution with the corrected BVR was selected as the final model and presented as the last model in Table 3.

**Table 3:** Class Enumeration for Step-1 of Latent Profile Analysis

Model	BIC (LL)	AIC (LL)	AIC3 (LL)	Max. BVR	Entropy R^2^	BLRT *p*-value
1-profile	3772.02	3734.46	3746.46	52.51	1	-
2-profile	3620.56	3542.32	3567.32	28.38	.81	.00
3-profile	3601.73	3482.80	3520.80	20.50	.84	.00
4-profile	3590.08	3430.46	3481.46	6.42	.86	.00
5-profile	3598.12	3397.80	3461.80	5.44	.86	.00
6-profile	3602.28	3361.28	3438.28	5.83	.88	.00
7-profile	3615.17	3333.47	3423.47	5.53	.89	.00
8-profile	3628.22	3305.85	3408.85	3.67	.92	.00
9-profile	3680.08	3317.01	3433.01	4.22	.91	.62
10-profile	3725.07	3321.32	3450.32	2.83	.90	.14
**4-profile BVR Corr**	**3503.18**	**3268.44**	**3343.44**	**1.87**	**.82**	-

The chosen model is presented in bolded italics. Fit was evaluated with the BIC, AIC, AIC3, and BVR

*Corr.* = correction, *LL* = log likelihood, *BIC* = Bayesian information criterion, *AIC* = Akaike information criterion, *AIC3* = Akaike information criterion 3, *Max.* = maximum, *BVR* = bivariate residual, *BLRT* = bootstrap likelihood ratio test

**Fig. 1:**
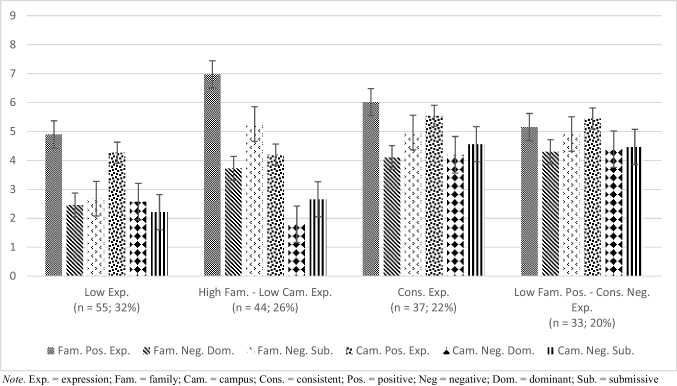
Raw Means for Profiles of Contextual Emotion Expression

**Fig. 2:**
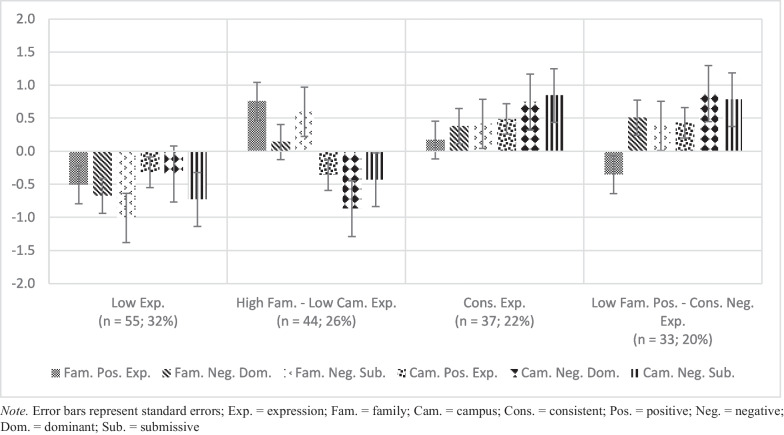
Standardized Means for Profiles of Contextual Emotion Expression

Profiles of contextual emotion expression are displayed by raw means of the expression variables in Fig. 1 and standardized means of the expression variables in Fig. 2.

Profile 1 (*n* = 55; 32%) is referred to as *Low Expressors*. Students in the *Low Expressors* profile reported the lowest frequency of emotion expression relative to the mean. Students in profile 2 reported more frequent levels of emotion in the family context and the lowest levels of emotion in the campus context compared to the sample; this profile is labeled *High Family – Low Campus Expressors* (*n* = 44; 26%). Profile 3 (*n* = 37; 22%), called *Consistent Expressors*, included students who expressed somewhat frequent emotion expression across contexts and above average levels of emotion relative to the mean. Lastly, students in profile 4 (*n* = 33; 20%), *Low Family Positive – High Campus Negative Expressors*, expressed the second lowest levels of family positive emotion and the highest levels of negative emotion on campus of all profiles. In summary, 4 profiles of emotion expression in the family and on campus emerged: 2 of which emotion expression was consistent across contexts and 2 of which emotion expression was generally different between family and campus but had some nuances in the frequency of expression according to the type of emotion.

In steps 2 and 3 of Vermunt’s approach to LPA, college campus racial composition type, racial discrimination, and racial composition of friend group were examined as covariates of profile membership. Posterior probabilities for profile membership were estimated during Step-2. Given the conclusion from Step-1, a 4-class solution was estimated in Step-2. Step-3 then estimated posterior probabilities with college campus type, racial discrimination, and racial composition of friend group as covariates. The first model only included college campus racial composition type and racial discrimination. According to Wald tests, campus racial composition type was not significantly related to profile membership, *χ*^2^ = 2.93, *p* =.82, yet racial discrimination was significantly related, *χ*^2^ = 7.75, *p* =.05. Post hoc paired comparison tests revealed that students in the *Low Expressors* (*n* = 55; 32%) profile experienced significantly different levels of racial discrimination than students in the *Low Family Positive – Consistent Negative Expressors* (*n* = 33; 20%; Wald = 7.30, *p* =.01) profile. More specifically, *Low Family Positive – Consistent Negative Expressors* (*n* = 33; 20%; M = 2.36) reported experiencing significantly higher levels of racial discrimination than *Low Expressors* (*n* = 55; 32%; *M* = 2.15). The second model with all three covariates revealed that racial discrimination and racial composition of friend group were significantly related to profile membership, respectively, *χ*^2^ = 7.05, *p* =.07 and *χ*^2^ = 8.31, *p* =.04. Post hoc paired comparison tests revealed that no significant differences emerged in racial discrimination experiences between profiles. However, *High Family – Low Campus Expressors* (*n* = 44; 26%) had significantly different amounts of Black people in their friend group than *Low Family Positive – Consistent Negative Expressors* (*n* = 33; 20%; Wald = 6.54, *p* =.01). Students in the *High Family – Low Campus Expressors* (*n* = 44; 26%; *M* = 1.83) profile had more Black friends than the Low *Family Positive – Consistent Negative Expressors* (*n* = 33; 20%; *M* = 2.74) profile. Overall, steps 2 and 3 of Vermunt’s approach to LPA revealed that profiles of African American college students’ emotion expression co-varied with racial discrimination experiences and racial composition of friend group on campus such that *Low Family Positive - Consistent Negative Expressors* reported higher levels of racial discrimination than *Low Expressors* and *High Family – Low Campus Expressors* reported more Black friends than *Low* *Family Positive – Consistent Negative Expressors*.

## Discussion

The overall goal of this study was to explore patterns of African American college students’ emotional expression and contextual predictors of these profiles as a potential reflection of cultural adaptation to relative threat in public (i.e., campus) versus private (i.e., family) contexts, campus-specific representation of their own and other racial groups, and experiences with racism on campus. We expected to see differential profiles of African American college students’ emotional expression across campus and family contexts and the modification of emotional expression according to such contextual features given theoretical notions of cultural and racial emotion messaging they may receive in the family (Boykin & Toms, 1985; Dunbar, et al., 2017). Four profiles total emerged: 2 of which emotion expression was consistent across contexts and 2 of which emotion expression was generally different between context and the type of emotion. Below, we discuss the profiles according to this interpretation.

### Profiles with Consistent Emotion Expression Across Context

Students in the *Low Expressors* profile reported little expression of emotion in the family and on campus, suggesting consistent emotion restriction across contexts. Approximately 1/3 of the sample was characterized in this profile, which was the largest of all profiles. Such a pattern of expression is consistent with earlier theorizing about high self-control and a reluctance toward self-disclosure of negative emotions among African Americans (Consedine & Magai, 2002; Plasky & Lorion, 1984). While it is unclear to what extent *Low Expressors'* emotion expression reflects that of their parents, it is possible that their overall family emotional climate is characterized by low levels of expressiveness (Halberstadt, 1986). Thus, the approach of *Low Expressors* may be safe in that maintaining a placid or stoic disposition allows them to conform to emotional norms across contexts (Hayes & Metts, 2008).

Students in the *Consistent Expressors* profile expressed average levels of emotion regardless of the type of emotion and progressively higher levels of emotion than the rest of the sample in both family and campus contexts. Twenty-two percent of participants were in the *Consistent Expressors* profile. The emergence of this profile suggests that some students in our study felt indifferent about the type of emotion they expressed (i.e., positive, negative), which contradicts hypothesis 1 in some ways. Perhaps parents of *Consistent Expressors* are generally supportive of emotion in the family, making the distinction of positive and negative emotion less salient.

### Profiles with Contextual Differences in Emotion Expression

The two profiles that reflected contextual differences were the *High* *Family – Low Campus Expressors* and the *Low Family Positive – Consistent Negative Expressors*. The *High Family – Low Campus Expressors* were characteristic of 26% of the sample and included students with more frequent family expression than campus expression for all emotions and infrequent campus expression altogether. It is worth noting that students in this profile reported the highest level of family positive emotion expression of all profiles, suggesting that their overall family emotional climate can be characterized as highly expressive. For this reason, it is interesting that *High Family – Low Campus Expressors*’ intensity of emotion expression did not translate to the campus context.

*Low Family Positive – Consistent Negative Expressors*, on the other hand, reported consistent levels of negative emotional expression across contexts, but particularly low positive emotion in the family, representing approximately 20% of the sample. Students in this profile appeared to have some comfort with expressing negative emotion regardless of the context, which makes the *Low Family Positive – Consistent Negative Expressors* profile unique in the sense that their pattern of emotion expression suggests a slight preference for negative emotion over positive emotion. Previous literature on African Americans’ sociocultural orientation toward emotion is mixed in that it suggests they value emotion expression and emotion-related information, broadly, and prefer positive emotion to negative emotion (Boykin, 1983; Labella, 2018), the latter of which is partially due to the communal aspect of Black culture (Halberstadt & Lozada, 2011). Our finding of the *Low Family Positive – Consistent Negative Expressors* profile complicates this literature further and suggests the need for additional work on African Americans’ emotion expression in public contexts. Taken together, the emergence of four profiles of college students’ emotion expression across family and campus contexts may illustrate the importance of social context for African Americans’ emotion-related behavior (Greenaway et al., 2018; Nelson, Leerkes, O’Brien et al., 2012). Our findings extend previous literature that primarily considers African Americans’ emotion expression in discrete contexts (Hill & Tyson, 2008; Nelson, O’Brien, Calkins et al., 2012; Vrana & Rollock, 1996, 1998, 2002) and demonstrate unique patterns of emotion-related behavior in public that may emerge in African American young adults’ navigation of multiple contexts (Boykin & Toms, 1985).

### Covariates of Profiles of Emotion Expression Across Context

We expected college campus type (i.e., PWI, racially diverse institution, HBCU) and racial discrimination experiences to be related to African American college students’ emotion expression profiles. This hypothesis was partially supported, as the type of college did not account for profiles of emotion expression in the family and on campus. One explanation for the null findings of school racial composition on profile membership is that other indicators of social context might have activated specific patterns of emotion expression for African American/Black college students (e.g., competition and relational closeness; Greenaway et al., 2018). It is also possible that students’ specific perceptions of the cultural values and emotional expectations in their various social contexts may be more strongly related to their patterns of emotion expression. Racial discrimination experiences, on the other hand, were related to profiles of Black students’ emotion expression in the family and on campus. Members of the *Low Expressors* profile were less likely to experience racial discrimination in comparison to the *Low Family Positive – Consistent Negative Expressors* profile. Our findings add to previous work with African American college students that found scenarios of both blatant and subtle experiences of racial discrimination to be predictive of various emotional responses (Jones et al., 2014). While our study design did not allow us to determine whether self-reported racial discrimination experiences by African American students on campus predicted their patterns of emotion expression across the family and campus context, we speculate about the meaning of the weaker association between racial discrimination and the *Low Expressors* profile relative to the *Low Family Positive – Consistent Negative Expressors profile*. When someone is low in emotional expression, one possibility is that they are masking their emotional experience. Another possibility is that they are not experiencing strong emotions. Perhaps the *Low Expressors* profile included subgroups of students with varying trait-like dispositions for emotional experience, some who feel emotions strongly and frequently and mask their display of emotions and others who do not experience intense emotions often.

Lastly, we explored the relationship between the racial composition of African American college students’ friend group and their patterns of emotion expression across family and campus contexts. We found that racial composition of friend group mattered for patterns of emotion expression such that *High Family – Low Campus Expressors* (*n* = 44; 26%; M = 1.83) tended to have more Black friends than *Low Family Positive – Consistent Negative Expressors* (*n* = 33; 20%; M = 2.74). In other words, the presence of Black people within students’ friend group was related to high levels of positive expression within the familial context and low expression of emotion in the campus context. There are several possibilities for what might contribute to this finding keeping in mind that we did not test for causation. One possibility is that *High Family – Low Campus Expressors* did not feel comfortable expressing their emotions on campus, which is at least associated with the racial demographics of students in this profiles’ friend group. Previous research on African American college students on a predominantly White campus has found that they are generally more expressive of positive emotion than negative emotion (Vrana & Rollock, 1996, 1998, 2002). Our finding held across college campus type and adds to literature on the role of context in African Americans’ emotion expression. Future work would need to introduce temporal ordering with racialized contextual factors and African American college students’ patterns of emotion expression to determine causality and perhaps see how students’ comfort with social relationships may be changing over time and potentially influencing their emotion-related behavior.

## Limitations, Future Directions, and Conclusions

The current study is not without limitations. First, the data was cross-sectional, so we cannot make any conclusions about causation between covariates and profiles of emotion expression among African American/Black college students. Second, our reliance on self-report measures may have contributed to mis- or under-reporting of contextual emotion expression. While the current study is unique in that it modified the instructions of the SEFQ, a validated and well-used measure of emotion expression within the family context, to ask participants how they express emotion on campus, there may be multiple contexts and relationships within campus settings—roommates, friends, classmates, and authority figures—which were not distinguished in our SEFQ modification. More nuanced consideration of campus contexts and observation of emotional expression with family members and public figures will be an important augmentation in future studies.

The current study extends knowledge of African Americans’ emotion expression by considering public contexts and patterns of contextual variation in expression across family and campus settings. This research provides an initial step in understanding the ways in which African Americans’ navigation of culture and race in the US may shape their emotion-related behaviors. Our findings suggest that African American emotional expressivity is not uniform; rather, African Americans display multiple patterns of emotion expression. Further research is needed to replicate and extend these findings by examining whether patterns denoting greater expressive flexibility across contexts are related to adaptive outcomes, as might be expected given the necessity of navigating multiple contexts for African Americans (Lozada, Jagers et al., 2022). The contextual study of emotion-related behaviors among African Americans will advance both affective science and prevention efforts to support African Americans’ emotional health and well-being.

## Footnotes

1 We use the term “Black” when referring to the cultural realm of experience based in West African cultural values as this is consistent with the terminology used in Triple Quandary Theory (Boykin & Toms, 1985) when describing the three cultural realms that African Americans must navigate. Otherwise, we use the terms African American or African American/Black (the latter when describing our sample) because this is the term we used on study recruitment materials and how participants self-identified on the study demographic questionnaire.

## Additional Information

### Data Availability

Study data, methods, and materials will not be made publicly available in a data repository, but a de-identified dataset for the current study will be made available upon request.
